# Early SARS-CoV-2 Reinfections within 60 Days and Implications for Retesting Policies

**DOI:** 10.3201/eid2808.220617

**Published:** 2022-08

**Authors:** Louis Nevejan, Lize Cuypers, Lies Laenen, Liselotte Van Loo, François Vermeulen, Elke Wollants, Ignace Van Hecke, Stefanie Desmet, Katrien Lagrou, Piet Maes, Emmanuel André

**Affiliations:** University Hospitals Leuven, Leuven, Belgium (L. Nevejan, L. Cuypers, L. Laenen, L. Van Loo, F. Vermeulen, I. Van Hecke, S. Desmet, K. Lagrou, E. André);; Rega Institute, Katholieke Universiteit Leuven, Leuven (E. Wollants, S. Desmet, K. Lagrou, P. Maes, E. André)

**Keywords:** COVID-19, SARS-CoV-2, reinfections, testing policies, epidemiology, vaccination, coronavirus disease, severe acute respiratory syndrome coronavirus 2, viruses, respiratory infections, zoonoses, Belgium

## Abstract

Illustrated by a clinical case supplemented by epidemiologic data, early reinfections with SARS-CoV-2 Omicron BA.1 after infection with Delta variant, and reinfection with Omicron BA.2 after Omicron BA.1 infection, can occur within 60 days, especially in young, unvaccinated persons. The case definition of reinfection, which influences retesting policies, should be reconsidered.

The sequential emergence of SARS-CoV-2 variants of concern (VOCs), characterized by an antigenic drift and higher transmissibility, has been observed in countries around the world at least 3 times during the past 13 months (*1*). Although the SARS-CoV-2 Delta variant showed a limited antigenic diversity with previous VOCs, Omicron differs more notably from other VOCs than any previous VOC did at the time it emerged (K. Van der Straten et al., unpub. data, https://www.medrxiv.org/content/10.1101/2022.01.03.21268582v2). The resulting decrease of antibody efficacy in both convalescent and vaccinees’ serum samples drives the high number of reinfection and vaccine breakthrough cases observed with Omicron compared with observations made during previous waves (*2*,*3*).

To date, reinfections with SARS-CoV-2 are defined by the European Centre for Disease Prevention and Control as a positive PCR or rapid antigen test >60 days after previous positive PCR, rapid antigen test, or serologic test (*4*). This definition has influenced testing strategies in several countries, and many countries consider a person protected for 180 days after an initial positive test result (*5*). We suggest that this reinfection definition should be revised.

To illustrate our point, we report a case of an immunocompetent unvaccinated 10-year-old boy with no noteworthy medical history who tested positive for SARS-CoV-2 Delta variant (>7.0 log copies/mL, sublineage AY.43) on December 3, 2021, concomitant with an outbreak at the patient’s school. The patient’s brother and mother, both vaccinated, were infected as well. All 3 persons experienced mild COVID-19 symptoms. Because of a sports-related trauma, the patient was admitted for surgery on January 11, 2022. Preprocedural SARS-CoV-2 screening detected a strong positive result (5.1 log copies/mL) with Omicron BA.1 variant, only 39 days after the patient’s infection with Delta. The patient remained pauci-symptomatic. High-risk contact screening of the patient’s brother detected a low viral load; the mother tested SARS-CoV-2–negative (Appendix Table).

To put this clinical case into a wider epidemiologic context, we estimated the incidence of early reinfection with Omicron BA.1 after Delta infection and reinfection with Omicron BA.2 after BA.1 infection in a community setting (Flemish Brabant, Belgium). During December 1, 2021–February 7, 2022 (n = 9 weeks), a period characterized by the full viral replacement of Delta by Omicron BA.1 (Appendix Figure 1) (*6*), a total of 59,515 ambulatory patients tested SARS-CoV-2–positive at the federal testing platform located in Leuven, Belgium (positivity rate 36.5%). Among these patients, the spike (S) gene was detected in a first sample in 0.15% (91/59,515 persons) by using the TaqPath PCR test (ThermoFisher Scientific, https://www.thermofisher.com), which suggests Delta infection. S gene target failure was reported in a second positive sample within this period (nucleocapsid gene cycle threshold <27.8), indicating a reinfection with Omicron BA.1 briefly after Delta infection (*7*).

Similarly, during January 1, 2022–March 10, 2022 (n = 9 weeks), a period characterized by the emerging viral replacement of Omicron BA.1 by BA.2 but declining disease prevalence (Appendix Figure 1) (*6*), a total of 58,166 patients tested SARS-CoV-2–positive (positivity rate 48.3%). Among these, 0.01% (5/58,166) demonstrated S gene target failure in a first sample but an S gene was detected in a second positive sample, indicating a reinfection with Omicron BA.2 after BA.1 infection in these patients.

We noted the age and vaccination status of these 96 patients with documented early reinfection and compared that with the vaccination rate for the same age groups in the same geographic region (Flanders, Belgium) (Figure) (*8*). Early reinfections were most frequently observed among young unvaccinated patients (<12 years of age). Compared with the corresponding age groups in the general population, patients with early reinfections tended to be unvaccinated, partially vaccinated, or vaccinated but not boosted. Median time between the 2 positive samples with different VOCs was 47 days (range 17–65 days) (Appendix Figure 2).

**Figure:**
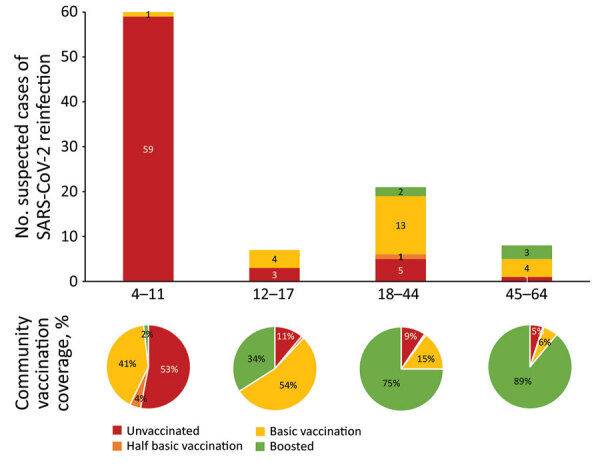
Number of patients with presumed SARS-CoV-2 reinfection including vaccination status compared with age-corresponding vaccination coverage in the community, Flanders, Belgium. Consecutive infections were detected during December 1, 2021–February 7, 2022 (reinfection with Omicron BA.1 shortly after Delta infection, n = 91 patients) and during January 1–March 10, 2022 (reinfection with Omicron BA.2 shortly after Omicron BA.1 infection, n = 5 patients). Half basic vaccination indicates 1 vaccine of ChAdOx1 nCoV-19 (AstraZeneca, https://www.astrazeneca.com), BNT162b2 (Pfizer-BioNTech, https://www.pfizer.com), or mRNA-1273 (Moderna, https://www.modernatx.com); basic vaccination indicates 2 vaccines of ChAdOx1, BNT162b2, or mRNA-1273 or 1 vaccine of Ad26.COV2.S (Johnson & Johnson/Janssen, https://www.janssen.com); boosted indicates basic vaccination followed by 1 vaccine of BNT162b2 or mRNA-1273.

Previous retrospective cohort studies (*2*) showing a prolonged maintenance of protection against reinfection should be questioned after the emergence of Omicron. Our data confirm that early Omicron BA.1 reinfection (<60 days) after Delta infection and BA.2 reinfection after BA.1 infection can occur, especially in young, unvaccinated persons. In older patient groups, unvaccinated persons and persons who had received basic vaccination but no booster might be more vulnerable to reinfections than patients who received a first booster vaccine. Data from Denmark (M. Stegger et al., unpub. data, https://www.medrxiv.org/content/10.1101/2022.02.19.22271112v1) suggest reinfection usually results in mild disease not requiring hospitalization, as demonstrated by the case we report here.

The occurrence of a full viral replacement in a matter of weeks will continue to affect the duration and efficacy of immunity in the future. For this reason, in cases of sustained variant circulation, indications for retesting persons after a previous SARS-CoV-2 infection within 180 days are limited. However, in cases of cocirculation or switch of VOC with antigenic drift within this period, this minimum retesting interval should be omitted to adequately detect SARS-CoV-2 reinfections.

This article was preprinted at https://www.medrxiv.org/content/10.1101/2022.04.04.22273172v1.

Appendix Additional information about early SARS-CoV-2 reinfections within 60 days and implications for retesting policies
