# Supplementary material for: Early SARS-CoV-2 Reinfections within 60 Days and Implications for Retesting Policies
Source: Emerg Infect Dis. 2022 Aug;28(8):1729–31. doi: 10.3201/eid2808.220617 (PMC9328893; doi:10.3201/eid2808.220617)
Supplement: Appendix — Additional information about early SARS-CoV-2 reinfections within 60 days and implications for retesting policies [file 22-0617-Techapp-s1.pdf]

Article DOI: https://doi.org/10.3201/eid2808.220617

Early SARS-CoV-2 Reinfections within 60 Days and Implications for Retesting Policies

Appendix

Appendix Table. Results of SARS-CoV-2 screening tests and vaccination status in reinfection patient and family, Belgium*

Date	Patient, 10 y	Brother, 13 y	Mother, 42 y	Father, 43 y
Vaccination status	Unvaccinated	2021 Aug BNT162b2, 2021 Sep BNT162b2	2021 Jun BNT162b2, 2021 Jul BNT162b2	2021 Feb ChAdOx1, 2021 May ChAdOx1, 2021 Nov BNT162b2
First infection				
2021 Dec 2		SARS-CoV-2 very strong positive, ≥7.0 log copies/mL, Delta (AY.43)	SARS-CoV-2 strong positive, ≥5–<7.0 log copies/mL, Delta (AY.43)	
2021 Dec 3	SARS-CoV-2 very strong positive, ≥7.0 log copies/mL, Delta (AY.43)			SARS-CoV-2–negative
2021 Dec 8				SARS-CoV-2–negative
2021 Dec 19	SARS-CoV-2 weak positive, <3.0 log copies/mL, untypable			
Second infection				
2022 Jan 11	SARS-CoV-2 strong positive, 5.1 log copies/mL Omicron (BA.1)			
2022 Jan 17	SARS-CoV-2–negative	SARS-CoV-2 weak positive, <3.0 log copies/mL, untypable	SARS-CoV-2–negative	
2022 Jan 21	SARS-CoV-2–negative			

*All samples were analyzed by using reverse transcription PCR. Whole-genome sequencing was performed on SARS-CoV-2–positive samples in the Belgian National Reference Center for Respiratory Pathogens.

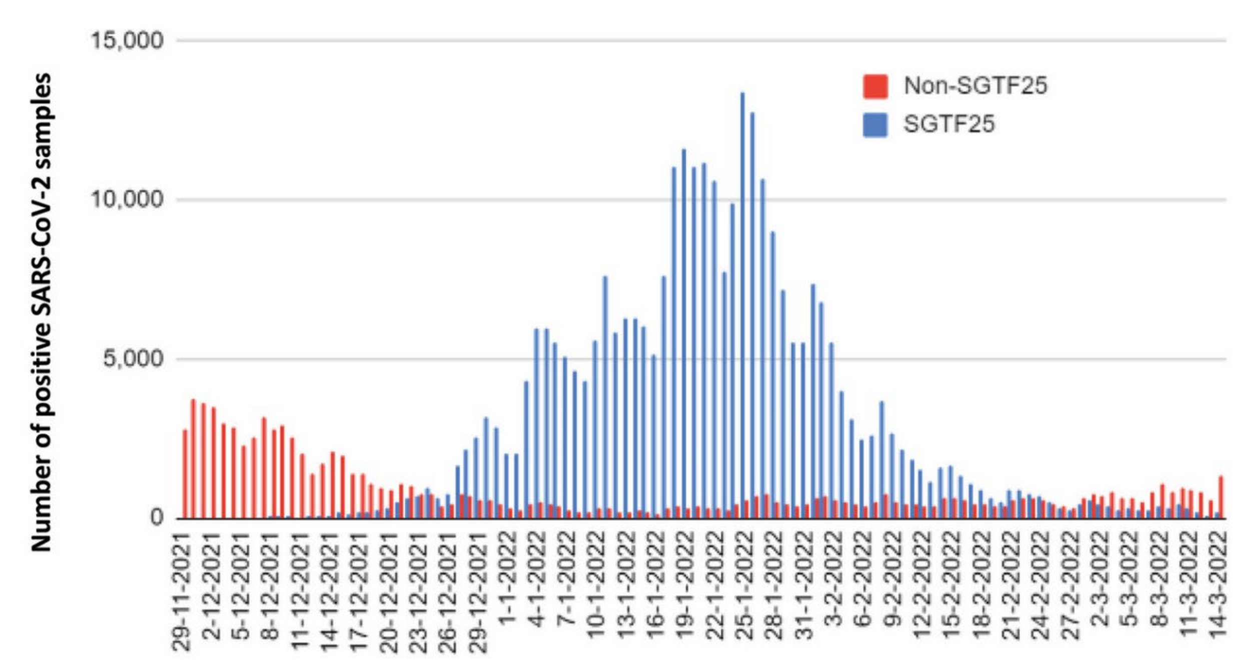

Appendix Figure 1. Number of samples testing SARS-CoV-2–positive in the federal platform laboratories of Belgium with SGTF (blue) and without SGTF (red) (*1*). SGTF, S gene target failure.

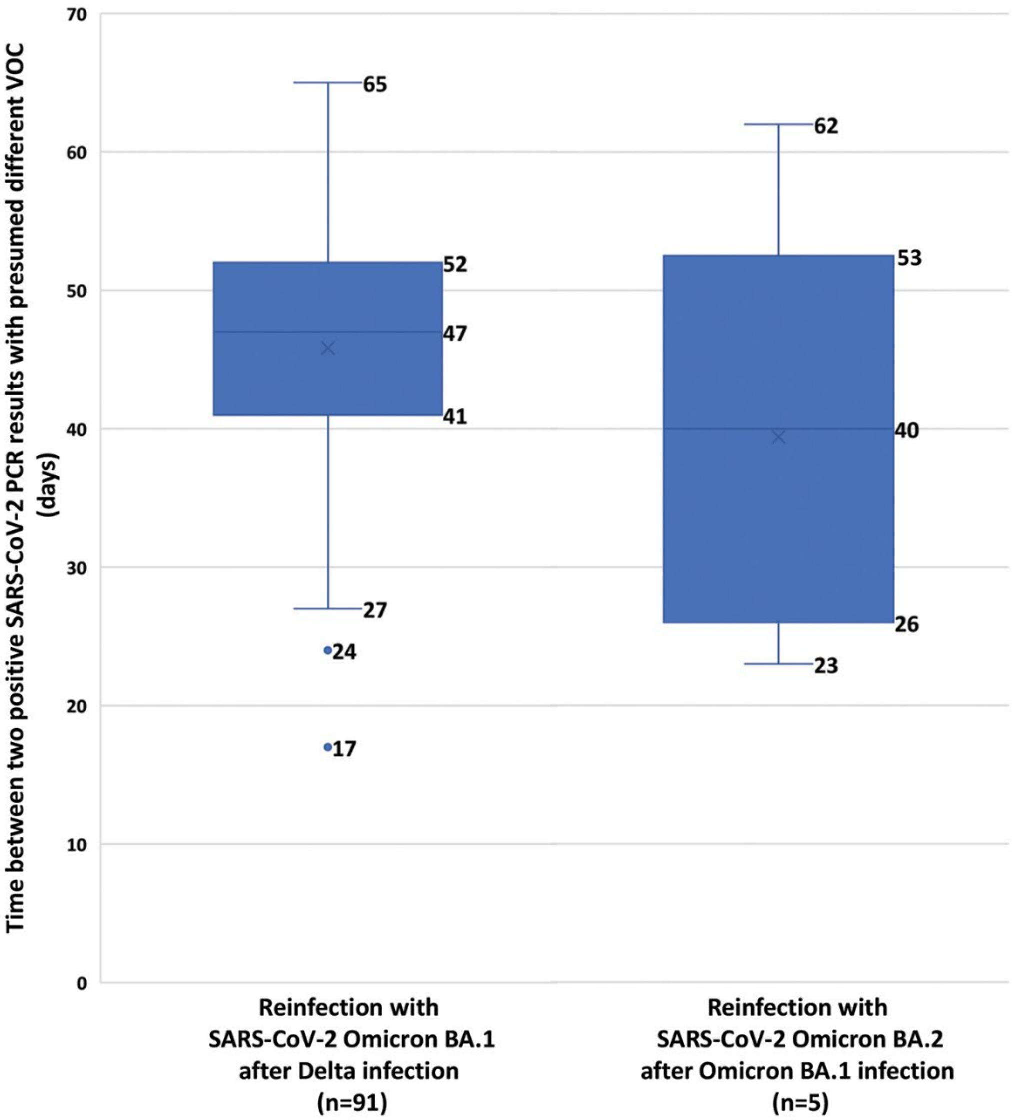

Appendix Figure 2. Boxplots indicating time in days between presumed SARS-CoV-2 infection and reinfection. The consecutive infections were detected during December 1, 2021–February 7, 2022 (reinfection with Omicron BA.1 shortly after Delta infection, n = 91 patients) and during January 1–March 10, 2022 (reinfection with Omicron BA.2 shortly after Omicron BA.1 infection, n = 5 patients). One patient had only 17 days between 2 positive SARS-CoV-2 PCR results with presumed different variants of concern. However, the first sample (2021 Dec 16, S gene detected) had a low viral load (<3.0 log copies/mL), the second sample (2022 Jan 2, S gene not detected) had a very strong viral load ($\geq$7.0 log copies/mL). As such, it can be stated that the time between the 2 positive samples was 17 days, but it cannot be assured when the Delta infection started, because viral remnant can be detected for several weeks after the initial infection and no clinical data was available for this case.

References

1. Cuypers L, Baele G, Dellicour S, Maes P, André E. Genomic surveillance of SARS-CoV-2 in Belgium [cited 2022 May 10]. https://www.uzleuven.be/nl/laboratoriumgeneeskunde/genomic-surveillance-sars-cov-2-belgium
